# A Multi-Tier Trust-Based Security Mechanism for Vehicular Ad-Hoc Network Communications

**DOI:** 10.3390/s22218285

**Published:** 2022-10-28

**Authors:** Brian Akwirry, Nik Bessis, Hassan Malik, Sarah McHale

**Affiliations:** Computer Science Department, Edge Hill University, Ormskirk L39 4QP, UK

**Keywords:** vehicle ad hoc networks, trust management, malicious attackers, VANET communication

## Abstract

Securing communications in vehicle ad hoc networks is crucial for operations. Messages exchanged in vehicle ad hoc network communications hold critical information such as road safety information, or road accident information and it is essential these packets reach their intended destination without any modification. A significant concern for vehicle ad hoc network communications is that malicious vehicles can intercept or modify messages before reaching their intended destination. This can hamper vehicle ad hoc network operations and create safety concerns. The multi-tier trust management system proposed in this paper addresses the concern of malicious vehicles in the vehicle ad hoc network using three security tiers. The first tier of the proposed system assigns vehicles in the vehicle ad hoc network a trust value based on behaviour such as processing delay, packet loss and prior vehicle behavioural history. This will be done by selecting vehicles as watchdogs to observe the behaviour of neighbouring vehicles and evaluate the trust value. The second tier is to protect the watchdogs, which is done by watchdogs’ behaviour history. The third security tier is to protect the integrity of data used for trust value calculation. Results show that the proposed system is successful in identifying malicious vehicles in the VANET. It also improves the packet delivery ratio and end-to-end delay of the vehicle ad hoc network in the presence of malicious vehicles.

## 1. Introduction

With the recent digitization, the connected vehicle ecosystem will soon be a reality, where vehicles will be communicating and exchanging information about each other and the environment [1,2]. This will lead to increased vehicle communication complexity and an expansion in the attack surface for VANET [3]. Therefore, there is a need to provide a secure mechanism through which such communication can take place. This work presents a multi-tier trust-based security mechanism in VANET to ensure a seamless and secure exchange of data among connected vehicles. The design of wireless communication technology and network systems is constantly evolving and progressing towards a better state, and vehicle ad hoc networks (VANETs) have gained considerable interest from researchers, automobile manufacturers and government institutions [4,5]. VANETs are a special type of mobile ad hoc network which enables communication on roads in modern environments [4,6]. By enabling communication, VANETs are able to provide real-time information such as traffic congestion warnings, safety messages, lane change information and infotainment [4,5,6]. This leads to optimized traffic conditions, increased road safety and improved driving conditions for road users [7]. Due to the importance of information transmitted in the VANET, accuracy and timely delivery of messages are crucial to gain the benefits mentioned earlier [8].

VANETs have enabled Vehicle-to-Vehicle (V2V) communication and cooperation, and have also been utilized in Vehicle-to-Infrastructure (V2I) contact [9]. V2V and V2I communication are the two main modes of communication performed by vehicles in a VANET [10,11]. V2V communication is made possible by the On Board Unit (OBU) present in most modern vehicles [12]. The OBU contains the GPS module, wireless communication module, Central Control Module (CCM) and human-machine interface module [13]. V2I communication is made possible by deploying Road Side Units (RSU) along roads or intersections [4]. V2I communication in some cases also involves communication with Trusted Authorities (TA) deployed along the route. TAs are a trusted third party deployed in VANETs equipped with networking features and computing power to manage the VANET [4]. Vehicles in the VANET communicate with other vehicles or RSUs by dedicated short-range communication (DSRC) on a single-hop or multi-hop basis [6,13].

One of the key challenges facing the implementation of VANETs is in providing secure vehicle communication [14]. Messages transmitted in the network must be guaranteed from modification, or insertion in the VANET. Security requirements for VANETs have been identified to be availability, integrity, confidentiality, authentication, non-repudiation, and traceability [6,13,15]. The high mobility, rapidly changing network topology, limited transmission power, volatility in network connections and boundless network size present a challenge for VANETs to achieve their security requirements [7,16]. These security aspects require different security mechanisms to achieve them.

Attacks in a VANET disrupt the normal working of the network and therefore lead to disruptions in the lifesaving aspect of the VANET. Attacks in the VANET can be performed by either an insider or outsider in the network [17]. Insider vehicles in the network are referred to as the vehicles that are authorised members of the network and can communicate with other vehicles in the network [16]. While outsider vehicles do not have direct access to the network and cannot communicate with members of the VANET [16]. Because the outsiders have limited access, they also have a limited capacity to attack the network, thus insider attacks are more dangerous. Some of the attacks that can be performed on VANETs include Sybil attack, Denial-of-Service attack (DOS), Distributed Denial-of-service attack (DDOS), Blackhole attack, Wormhole attack, Message suppression attack, Message Alteration attack, Replay attack, Timing attack, Man-in-the-middle attack and Eavesdropping attack [7,16,17,18,19].

Cryptography secure systems for VANETs have been found to be ineffective for securing the VANET from malicious vehicles and attacks [1,20]. Some of the weaknesses identified in cryptography solutions are the inability to deal with the dynamic and distributed nature of vehicle networks. Cryptography systems have also failed in dealing with insider attacks, which are the most dangerous type of attacks in VANETs.

Trust management systems have the ability to fill the gap of providing security in ad hoc networks. Trust management has been found to be a good solution to handle internal attackers if executed correctly [21]. Trust management systems can enable vehicles to cooperate within the network and avoid vehicles exhibiting malicious behaviour [22].

### 1.1. Motivation and Contributions

To the best of the authors’ knowledge, there is still a gap in designing an efficient trust management system for VANET. As most of the works presented in the literature consider real-time vehicle behaviour and ignore the vehicle behavioural history, or ignore the security of watchdogs or the integrity of the trust value calculation, which results in a high risk of false alarms and degradation of system performance. Therefore, this work presents an efficient trust management system based on vehicle behaviour for the detection of malicious vehicles and to improve security within VANET. The proposed system in this work considered federated resource management in the design and includes the vehicle behaviour history and integrity of data while calculating trust values of the vehicles. The overall calculation of trust value is done at RSU, which is more resourceful, and watchdogs are used for forwarding the data. The proposed system is a highly effective multi-tier trust management system that can identify malicious and non-malicious vehicles in a VANET. While being robust in functionality, the trust management system will remain simple, fast, and efficient. The proposed system will also protect against malicious watchdogs that may have been selected in the VANET. The main contributions of the work include:

- Proposed a multi-tier trust-based security mechanism based on vehicle behaviour.
- Proposed a security mechanism for protecting data integrity within the defined requirement of trust management in VANET communication.
- Proposed a security scheme to protect against malicious watchdogs in the VANET.
- Extended the proposed model to a multi-vehicle scenario providing a comprehensive review of the system with critical VANET factors, PDR and delay.

### 1.2. Paper Organization

The structure of this paper is as follows: Section 2 will give a detailed account of the related literature of the study. Section 3 gives a detailed discussion of the proposed system. This includes system requirements, components that make up the system, and the process of trust calculation. Section 4 illustrates the performance evaluation of the system, as well as the experimental results. Section 5 and Section 6 conclude the paper and present a discussion into the future work.

## 2. VANET Overview

Security is a major issue in VANET communication because the vehicles are exchanging sensitive information about themselves and their surroundings [23]. Securing communications is crucial for VANET operations. The following have been identified as characteristics that VANETs must satisfy to be secure in communication:

- Availability: In VANET communication, real-time data is used for many purposes, therefore the data must be available and accessible when needed [24]. Applications of VANET communication require a quick reaction to the data provided, therefore if there is any hold-up in the data, even for a few seconds, then the data could be rendered worthless.
- Authentication: This provides a guarantee that the data generated and forwarded by vehicles in the network are done by an authentic vehicle [25]. In VANET communication, it is especially important that the data are generated from an authentic vehicle because vehicles in the network react to the data they receive.
- Integrity: This ensures the data at the recipient and sender are the same and that data are only altered by authorized vehicles [13].
- Non-Repudiation (NR): The purpose of this is to avoid vehicles identified as malicious from refusing the offences [13,24]. Senders of messages cannot deny being the sender. Once a vehicle has been correctly identified as malicious it cannot masquerade as an innocent vehicle and transmit packets in the VANET.
- Confidentiality/Privacy: This gives a guarantee that the data will only be accessed by the authorised vehicles and that vehicle privacy will be maintained [6].

These characteristics make VANETs vulnerable to malicious activity. The following section shall look at some of the attacks that can be propagated in VANETs.

### 2.1. Attacks in VANETs

Malicious vehicles in the VANET threaten the security of the VANET by deploying attacks. Attacks refer to malicious activity that is meant to cause harm to the system. The main idea behind executing these attacks is to intercept the messages and drop them or modify them for their own selfish purposes [7]. VANET communications are vulnerable to attacks because of the high mobility with frequent disconnections. Interactions and communications in the VANET only last for a limited amount of time [7]. These attacks tarnish the security requirements of vehicle-to-vehicle communications. The following have been identified as some of the attacks that can be propagated against VANETs.

- Black hole attack—In this attack, a malicious vehicle will claim to have the shortest route to a destination in the VANET [26]. The source vehicle will send the packets to the malicious vehicle, which will drop the packets instead of forwarding them to the intended destination [6,26].
- Wormhole attack—This attack is similar to a black hole attack, but is performed by two cooperating malicious vehicles. The malicious vehicles will form a tunnel, transmitting messages to the other malicious vehicles at the end of the tunnel, thus never forwarding to the intended destination [12,27].
- Message suppression/Alteration attack—In this attack, the malicious vehicles will either suppress the message by dropping it or alter the message to fulfil their agenda [6].
- Replay attack—In this attack, the malicious vehicle will receive a message and store the message instead of forwarding it to the destination [18]. The main purpose of the attack is to delay the message and replay it later, therefore delaying the effect of the message [4,28].
- Timing attack—The malicious vehicle in this attack will add delays to the message without altering the content of the message [14].
- Man-in-the-middle attack—In this attack, the malicious vehicle positions itself between two communicating vehicles, to gain access to the messages [7]. The malicious vehicle can alter the messages without the knowledge of the communicating vehicles [17].
- Eavesdropping attack—In this attack, the malicious vehicle will intercept and examine messages without altering the messages [18]. The main purpose of the attack is to gather information in preparation for a further devastating attack.

Table 1 shows a summary of attacks and their effects on messages in the VANET.

Due to the unique characteristics of VANETs, traditional security mechanisms cannot be used, and new security schemes had to be developed. Authentication of vehicles in the VANET is an integral step because it can be used by vehicles before accessing or sending messages and can prevent malicious vehicles [29,30]. Proper authentication schemes have the ability to easily identify malicious vehicles and illegitimate messages, therefore providing security in the VANET. Cryptography as an authentication scheme has shown a great ability to prevent external attacks, but not as efficient in insider attacks [20,30]. Although current trust management systems can handle internal attacks, improvements can be made to the systems to make them more effective and efficient. The next section shall look at the recent trust management systems developed, highlighting their advantages and disadvantages.

### 2.2. Related Work

A trust management system using two concepts, reputation and trust, was presented in [31]. Reputation was used to refer to the quantitative representation of the trustworthiness of a vehicle. This reputation will change depending on the behaviour of a vehicle. Trust in their scheme refers to the trustworthiness of the messages sent by the vehicles in the VANET. While [32] in their trust management scheme worked by estimating the trust level of a vehicle based on the opinions of its neighbouring vehicles. The trust management system in [21] assumes that vehicles can only have two levels, trusted and untrusted, and each time a vehicle is evaluated, it is taken as an independent process. Blockchain technology has also been integrated with trust management systems in VANETs [33,34,35,36]. BARS is an example of a blockchain-based reputation system for trust management suggested by [35], it makes use of the blockchain network instead of a central trust management system. Their trust management system consists of a punishment and reward mechanism. Trust management systems utilize selected vehicles to monitor other vehicles in the VANET [1,37,38,39,40], either as neighbour vehicles, watchdog vehicles, or cluster heads. Trust management systems must protect against vehicles that perform monitoring tasks in security solutions. It has been noted that reducing network overhead, low-latency communication, and intelligent resource management can be extremely challenging within a VANET context [1]. Energy and battery management is also one of the major challenges facing recently developed vehicles [41,42,43,44,45]. Therefore, efficiency must be considered in trust management design in order not to overwork the computational resources of a VANET. A range of recently designed security management systems for VANETs contain complex formulations that do not consider efficiency in their design. Complex formulations include systems that are computationally expensive that may decrease the communication efficiency of the VANET. Increased computational cost also leads to increased energy consumption by vehicles in the VANET [46]. Complex formulations such as formulations that make use of Rivest–Shamir–Adleman (RSA) have been found to be computationally expensive [27]. Formulations that make use of security, distribution and management of keys can also increase the complexity of a system [47]. However, its noted efficiency can be increased by distributing loads between vehicles [47]. Furthermore, if vehicles are responsible for monitoring and analysing vehicle behaviour, this leads to increased resource consumption. Consequently, this creates additional overheads for the vehicles because the monitoring of other vehicles in the VANET already consumes additional resources [48,49]. Vehicles with additional tasks such as analysing behaviour to distinguish malicious vehicles add additional computational and storage overheads [50,51]. The development of the proposed system considers intelligent resource management in its design. It will identify honest and malicious vehicles without draining computational resources in the VANET. The trust management systems were developed to make use of neighbour vehicles to monitor the VANET. However, the selected vehicles are not monitored during trust management operations. A malicious vehicle is selected to monitor other vehicles, which decreases the effectiveness of the trust management system. A variety of recently developed trust management systems do not check the integrity of data used to calculate the maliciousness or non-maliciousness of a vehicle. This can lead to inaccurate results in the VANET.

## 3. Proposed Trust Management System

Trust management systems have been proposed as a viable solution against malicious vehicles in a VANET. Trust management systems can be designed for various applications. The proposed trust management system was designed to identify malicious vehicles that drop messages instead of forwarding to the destination, and malicious vehicles that delay messages before forwarding to the destination. These were identified as the optimal metrics to identify attacks, as several malicious attacks cause the effect of vehicles dropping or delaying packets in the VANET as shown on Table 1. However, because the two metrics are dependent on network conditions, considerations had to be put in place. The proposed system will consider an additional metric of vehicle history, which will represent the vehicle behaviour from previous communication rounds. Unstable network conditions can cause vehicles to drop or delay messages despite the vehicles being non-malicious. This causes the identification of non-malicious vehicles as false positives in the VANET. The proposed system provides the identification of false positives for these scenarios as shown in the Section 4. The authors believe that the proposed system may not be effective against certain attacks such as location spoofing attacks, but the algorithm can be tailored for such applications.

### 3.1. Components of the Trust Management System

The proposed trust management system will be made up of the components described below.

- Vehicles—These are the vehicles that belong to the VANET. They are able to communicate with other vehicles in the VANET as well as the infrastructure.
- RSU—This provides a secure infrastructure component of the VANET. The RSU is assumed to be trusted, and highly resistant to attackers. For this reason, the security of the RSU is not considered in this work. The RSU is also responsible for the identification of malicious vehicles in the VANET, it will keep a record of malicious and non-malicious vehicles.
- Trust messages—Lightweight messages used to create data on vehicles in the VANET. They can only be created by vehicles with the watchdog agent activated.
- Watchdog agent—This agent can be applied to vehicles to enable monitoring mode. The watchdog agent is responsible for monitoring vehicle data and sending the data to the RSU. The watchdog collects data from readily available network information. In case a vehicle has recently joined the VANET and information is not available, the watchdog agent will forward trust messages in order to create data on the vehicle. Only verified trusted vehicles are selected as watchdogs in the VANET and only watchdogs are allowed to monitor data on vehicles. This significantly reduces the risk of a vehicle bad-mouthing another vehicle in the VANET.
- Threat agent—This agent can be applied to vehicles to enable malicious behaviour during VANET operations. The threat agent has access to vehicle communications and can control messages received by a vehicle. The threat agent can cause vehicles to drop messages received, delay messages received or both delay and drop messages received. This will simulate malicious behaviour in the VANET.
- Trusted vehicles—This is the set of vehicles that have not been taken over by the threat agent. They perform normal communication in the VANET.
- VANET—The VANET can exist in three states. In the first state, the VANET is run with no malicious vehicles present. This is used to create a baseline of the VANET when running in optimal conditions. In the second state, the VANET is populated with malicious vehicles, this indicates VANET behaviour in the presence of malicious vehicles. The third state involves applying the proposed system to a VANET with malicious vehicles present. The purpose of this is to evaluate the performance of the proposed system in a VANET made up of malicious vehicles.

### 3.2. Trust Management Functions

The trust management system will be designed to work within a VANET. In this particular scenario, a VANET is made up of autonomous vehicles. Multiple autonomous vehicles exist within an area, communicating with each other and the roadside unit. Among these vehicles, at least two trusted vehicles are selected, and the watchdog agent is activated in the vehicles. The function of these watchdogs is to monitor other vehicles in the VANET by collecting vehicle metrics. In order to minimize the overhead costs, the proposed system takes advantage of the information exchange that takes place via Internet Control Message Protocol (ICMP) requests by using data that are readily available in the VANET. In case the data are not available for a certain vehicle, the watchdog will send a trust message to the vehicle and collect the data from the message. The trust messages are designed to be small and lightweight in order to minimize the overheads incurred by the trust management system. The watchdog will send a trust message to a destination via an evaluated vehicle, and this evaluated vehicle will return an acknowledgement confirming the successfully delivery of the trust message. The watchdog is able to collect the required data from this transaction. The watchdogs accumulate the collected data and send it to the roadside unit. By sending the data to the roadside unit, it ensures fairness and no bias by the watchdogs in the VANET. The roadside unit performs the processing and calculation of a trust value from the data received by the watchdog. The trust value of a vehicle indicates its non-maliciousness or maliciousness. The trust value is calculated by considering the packet delivery ratio (PDR), processing delay (PD) and history of the vehicle. This will be discussed in further detail in the below section. The trust value lies between 0 and 1, a value closer to 0 indicates maliciousness while a value closer to 1 indicates non-maliciousness of the vehicle. The trust value is dynamically updated to match the vehicle’s behaviour at any given time. A trust threshold between 0 and 1 must be introduced to distinguish the minimum trust value a vehicle must have to be considered trustworthy. The threshold can depend on the VANET application, with applications requiring higher security, e.g., military applications having a higher threshold. The roadside unit calculates these trust values via the following mathematical concepts for each vehicle in the VANET.

### 3.3. Trust Management Architecture

In this particular scenario, a vehicle network has been applied to an area (A). Where multiple vehicles $\left(V_{n}\right)$ are randomly distributed such that a set of vehicles:

$$V_{n}\;where,n=\left\{1,2,3,\ldots \ldots ,N\right\}\;and\;N\;\in \;N$$

These vehicles are communicating with each other and a set of roadside units $\left(R_{s}\right)$:

$$R_{s}\;where,s=\left\{1,2,3,\ldots \ldots ,S\right\}\;and\;S\;\in \;N$$

A set of at least 2 watchdogs $\left(V_{n}^{\prime }\right)$ exist within the VANET such that:

$$V_{n}^{\prime },\;where,n=\left\{1,2,\ldots ,\ldots N\right\}\;and\;V_{n}^{\prime }\;\in \;V_{n}\;and\;N\in N$$

For trust evaluation, the proposed system has considered: packet delivery ratio, message integrity, history and consistency factor. The selected metrics are monitored from the vehicles by the vehicle watchdogs before sending them to the central watchdog to calculate the trust value. The calculation for the trust metrics is performed by the equations and algorithms presented below.

#### 3.3.1. Packet Delivery Ratio

The PDR aims to calculate the ratio of packets successfully delivered by a vehicle. It is calculated by the ratio of packets received by a vehicle, to the number of packets successfully forwarded by the vehicle. The PDR will be calculated by monitoring the number of acknowledgements $\left(A_{x}\right)$ and trust messages $\left(T_{y}\right)$ exchanged between vehicles $\left(V_{n}\right)$ in the VANET. The PDR of $\left(V_{n}\right)$ is calculated as:

(1) $$PDR\left(V_{n}\right)=\sum_{n}^{N}\frac{A_{x}}{T_{y}}$$

where:

x={1,2,…,X},y={1,2,…,Y},n={1,2,…,N}andX,Y,N∈N

#### 3.3.2. Processing Delay

This is the time an intermediate vehicle (evaluated vehicle) takes to process a packet before forwarding it to the destination and receiving it from the source. This is necessary to find out if the intermediate vehicle is tampering with data with additional information or any activity before forwarding it. It is calculated by finding the difference between the time an evaluated vehicle receives a packet from the source (γ) to the time it forwards the packet to the intended destination (λ). The processing delay (PD) of a vehicle $\left(V_{n}\right)$ is therefore calculated using the following equation:

(2) $$PD\left(V_{n}\right)=\sum_{n}^{N}\frac{\lambda_{i}-\gamma_{j}}{i}$$

where:

i={1,2,…,I},j={1,2,…,J},n={1,2,…,N}andI,J,N∈N

#### 3.3.3. Trust Value Calculation

The PDR and processing delay are integrated to form a trust value using the equation described below. Two weights are introduced, weight of PDR (β) and weight of processing delay (θ) where, β+θ=1. The purpose of the weights is that they can be adjusted depending on the application. If the application is more concerned about the number of packets being delivered, the weight (β) can be increased. Otherwise, if the application is concerned about the packets being altered, the weight (θ) can be increased. Under normal conditions, both (β) and (θ) equal to 0.5.

(3) $$TV\left(V_{n}\right)=\beta \times PDR_{V_{n}}+\theta \times PD_{V_{n}}$$

#### 3.3.4. Vehicle History

The vehicle history involves considering the previous trust value of a vehicle. This ensures the vehicle has to constantly exhibit non-malicious behaviour to be considered a non-malicious vehicle in the VANET. In the case a vehicle does not have a history, it is ignored during the first round of communication until a history is created. The previously recorded trust value ($\omega (V_{n})$) is combined with the newly calculated trust value ($TV(V_{n})$) using the equation described below:

(4) $$TV\left(V_{n}\right)=\frac{\omega_{V_{n}}+TV_{V_{n}}}{2}$$

The proposed system makes use of at least two watchdogs, therefore each watchdog $\left(V_{n}^{\prime }\right)$ in the VANET will calculate a trust value $\left(TV\left(V_{n}\right)\right)$. The following trust matrix is created for every $\left(V_{n}^{\prime }\right)$ in the VANET:

$$TV_{M}\left(V_{n}\right)=\left[\begin{array}{c} TV_{V_{n}^{\prime }}\\ \ldots \\ TV_{V_{N}^{\prime }} \end{array}\right],where,n=\left\{1,2,3,\ldots \ldots ,N\right\},N\in N$$

The trust values from different watchdogs must be integrated to form a value that will represent the trust of a vehicle. This is done using the following equation:

(5) $$TV\left(V_{n}\right)=\frac{\sum_{n}^{N}\left(TV_{V_{n}^{\prime }}\right)}{N}$$

where:

n={1,2,……,N}andN∈N

This $TV(V_{n})$ represents the trust value of a vehicle $V_{n}$ in the VANET. This represents the behaviour of a vehicle.

#### 3.3.5. Trust Threshold

The calculation of $TV\left(V_{n}\right)$ in (4) is compared with the selected application trust threshold as presented in (6) below. This will distinguish between the malicious and trusted vehicles in the VANET.

(6) $$TV\left(V_{n}\right)>threshold$$

The proposed system makes use of defined controls to ensure data integrity, which are discussed below.

### 3.4. Data Integrity

The trust value is used to define the behaviour of a vehicle; therefore, this value is principal to the trust management system and VANET as a whole. Therefore, the integrity of the trust value must be protected against maliciousness. Controls have been proposed and implemented by the trust management system to protect the trust value. The controls ensure that data used to calculate the trust value is legitimate data and not fabricated by a vehicle or watchdogs. The first control is applied as (7) before Equation (1) is processed. This control ensures that the total number of acknowledgements is never greater than the total number of trust messages sent. This control is based on the fact a vehicle can only create acknowledgement messages after forwarding a message successfully. Therefore, the total number of messages forwarded should always be greater or equal to the total number of acknowledgements received. If the total number of acknowledgements received is greater than the total number of messages, the vehicle can be said to be fabricating acknowledgement messages therefore malicious. The control equation is described below:

(7) $$\sum_{n}^{N}V_{n}\left(T_{y}\geq A_{z}\right)$$

The second control is implemented as (8) before Equation (2) is executed. This control checks that the acknowledgement timestamp is always greater than the trust message time stamp. The acknowledgement timestamp should always be greater than the trust message timestamp. If the acknowledgement time stamp is less than the message forwarded time stamp, the vehicle can be considered to be fabricating data and considered malicious. The control equation is presented below:

(8) $$\sum_{n}^{N}V_{n}\left(\lambda_{i}\geq \gamma_{j}\right)$$

The third control applied via (9) is used to confirm the integrity of the data collected by the watchdogs in the VANET. This is done by comparing the data collected by the different watchdogs. The data collected by the watchdogs about an evaluated vehicle $V_{n}$ should be correlated and similar, as it was collected under similar conditions. The third control is applied after the trust matrix ($TV_{m}$) is calculated. The trust values from different watchdogs are compared as shown below:

(9) $$for\int_{n}^{N}\overset{is}{\to }TV_{V_{n}^{\prime }}==TV_{V_{N}^{\prime }}$$

The equations are made use of in the algorithms in order to enable the proposed system to function. The main purpose of Algorithm 1 is to calculate a trust matrix about the evaluated vehicle in the VANET. This assists the vehicle in achieving its objective function of being able to identify non-malicious and malicious vehicles.

The main purpose of Algorithm 2 is to integrate the trust of the evaluated vehicle from the trust values calculated by the watchdogs in the VANET.

The simulation was run assuming a dynamic topology in the network. The proposed trust management took advantage of a cluster formation in order to evaluate vehicles in the VANET. A cluster is made up of vehicles and infrastructure. The vehicles will include vehicles selected as watchdogs and the vehicles that will be evaluated. The infrastructure in the cluster will be made of roadside units. The cluster formation should have at least two watchdogs present in the VANET for the proposed trust management system to function optimally. There is no upper limit to the number of watchdogs required in the VANET. The proposed system is designed to work in areas where vehicles experience low speed such as parking lots and drive through restaurants. Additional simulation parameters are presented in Table 2.
**Algorithm 1: **Calculating Trust value matrix $\left(TV_{m}\right)$**Input:** Vehicle map: $(V_{n},R_{s}),\beta ,\theta$**Output:**$\left(TV_{m}\right)$ for every $\left(V_{n}\right)$**while **t∈T** do**    **$TV_{M}$:**    Select $V_{n}^{\prime }$ from $V_{n}$    //$V_{n}^{\prime }$ collect data on $V_{n}$    //$V_{n}^{\prime }$ forward data to $R_{s}$    **if** $\sum_{n}^{N}V_{n}\left(T_{y}\geq A_{z}\right)$ **then**        calculate $PDR(V_{n})$ by Equation (1)    **end if**    **if** $\sum_{n}^{N}V_{n}\left(\lambda_{i}\geq \gamma_{j}\right)$ **then**        calculate $PD(V_{n})$ by Equation (2)    **end if**    **for** $V_{n}^{\prime }\in V_{n}$ **do**        update trust matrix $TV_{M}\left(V_{n}\right)$ by Equation (3)    **end for****end while**

**Algorithm 2: **Calculating Trust Value ($TV(V_{n})$)
**Input:** Vehicle map: $V_{n},R_{s},\omega ,$
**Output:** $TV\left(V_{n}\right)$


**$TV(V_{n})$:**

**for** $TV_{m}(V_{n})$ **do**
    **if** $TV\left(V_{n}\right)$ exists in database **then**        update label to $\omega (V_{n})$    **end if**    **if** $TV_{V_{n}^{\prime }}==TV_{V_{N}^{\prime }}$ **then**        calculate $TV(V_{n})$ by Equation (5)    **end if**    **if** $\omega (V_{n})$ exists in the database **then**        calculate $TV(V_{n})$ by Equation (4)    **end if**
**end for**
update $TV\left(V_{n}\right)$


**Figure 1:**
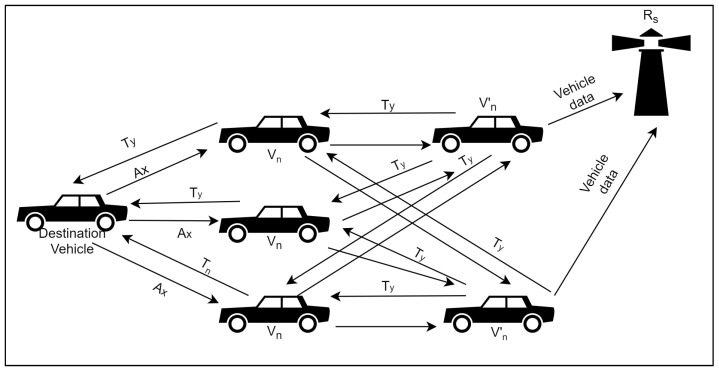
Simulation scenario showing operation of the proposed system.

The VANET architecture and communication can be seen in Figure 1. If data is readily available on the vehicles $\left(V_{n}^{\prime }\right)$, the watchdogs $\left(V_{n}^{\prime }\right)$ will collect this data and send it to the roadside unit $\left(R_{s}\right)$. In case data is not available, $\left(V_{n}^{\prime }\right)$ send trust messages $\left(T_{y}\right)$ to the vehicles $\left(V_{n}\right)$. $\left(V_{n}\right)$ will forward the messages to the destination vehicle which will send back an acknowledgement $\left(A_{x}\right)$ on receipt of $\left(T_{y}\right)$. $\left(V_{n}^{\prime }\right)$ monitor these transactions and send vehicle data to the roadside unit $\left(R_{s}\right)$.

## 4. Results

To evaluate the performance of the proposed system, the OMNET++ simulator was utilized for this. The proposed system is evaluated to prove functionality. Several different scenarios are applied to the proposed system including malicious vehicles that are dropping packets, delaying packets and scenarios with malicious vehicles both delaying and dropping packets.

Malicious behaviour will be simulated via the threat agent in randomly selected vehicles in the VANET. This will be used to evaluate the proposed system’s ability to identify malicious behaviour in vehicles. Three types of malicious vehicles will be simulated in the VANET.

- Malicious vehicles that drop messages—These malicious vehicles will receive messages from the source but will drop the messages instead of forwarding the messages to the destination vehicle. The vehicles will be simulated to drop messages at different rates within VANET operations. These will represent the following attacks that may cause messages to drop in a VANET: DOS attack, DDOS attack, blackhole attack, wormhole attack and replay attack.
- Malicious vehicles that delay messages—These malicious vehicles will receive messages from the source and instead of forwarding the messages directly to the destination vehicle, they will delay the message for a certain amount of time before forwarding the message. The vehicles will be simulated to delay messages at different rates in the VANET. These vehicles can be used to represent the following attacks that may cause delays in messages transmitted in a VANET: DOS attack, DDOS attack, message suppression/alteration attack, replay attack, timing attack, man-in-the-middle attack, and eavesdropping attack.
- Malicious vehicles that both delay and drop messages—These malicious vehicles will have the behaviour of vehicles that both delay packets and drop packets. They will both drop and delay messages at different times and at different rates during VANET operations. These vehicles simulate multiple attacks that may happen to a vehicle.

**Figure 2:**
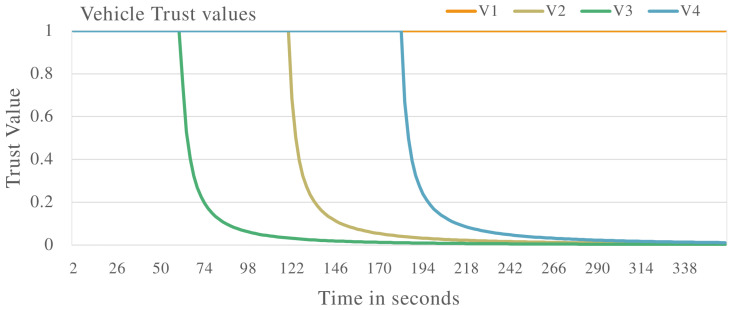
Vehicle trust values with malicious vehicles present that are dropping messages in the VANET.

The first experiment involves applying the proposed system to a VANET made up of vehicles exhibiting malicious and non-malicious behaviour. Malicious behaviour will involve the vehicle dropping messages at different rates in the VANET. Figure 2 shows the results of four evaluated vehicles, V1, V2, V3 and V4. V1 is identified as exhibiting non-malicious behaviour as its trust value is constantly at 1.0 throughout the VANET operation. V3, V2 and v4 are considered to be exhibiting malicious behaviour as their trust value drop through the VANET operation. The vehicles are identified as to be dropping packets during VANET operations. This shows the proposed system is successful in identifying non-malicious and malicious vehicles when malicious vehicles exhibit the behaviour of dropping packets.

**Figure 3:**
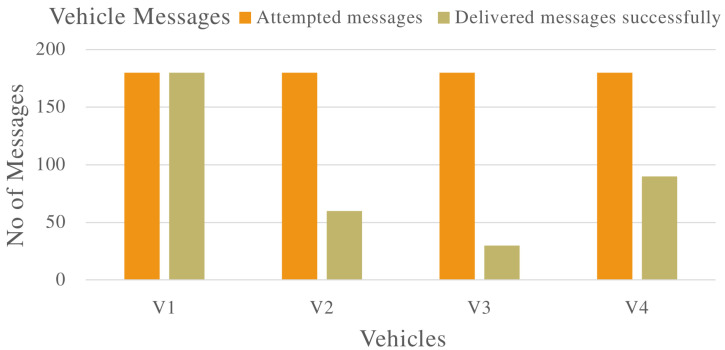
Total messages transmitted by vehicles in the VANET populated with malicious vehicles that are dropping packets.

The number of messages received and successfully forwarded to the destination by individual vehicles is shown in Figure 3. This further confirms the vehicles behaving suspiciously were dropping messages in the VANET.

**Figure 4:**
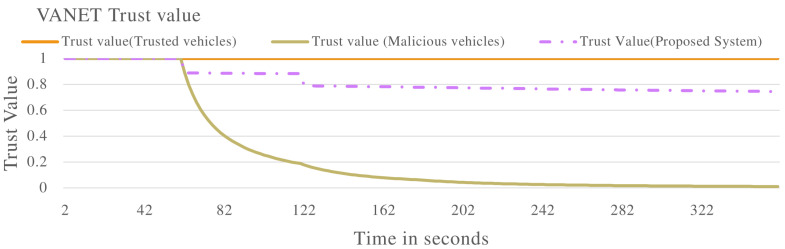
VANET trust value in multiple scenarios including malicious vehicles that are dropping packets in the VANET.

Figure 4 shows the overall trust value of the VANET. The blue line, trust value (Trusted vehicles), represents the trust value when the VANET is populated by all the vehicles exhibiting non-malicious behaviour. The trust value remains constant at 1.0. The orange line, the trust value (Malicious vehicles) line, shows a declining trust value until a level close to 0.0. This indicates the VANET has been taken over by malicious vehicles until a point it cannot perform its normal functions. The grey line, trust value (Proposed system), represents the proposed system when applied to the VANET with vehicles exhibiting malicious behaviour. The proposed system isolates malicious vehicles, thereby stopping the malicious vehicles from taking over the VANET. The VANET can therefore perform its normal functions even in the presence of malicious vehicles.

**Figure 5:**
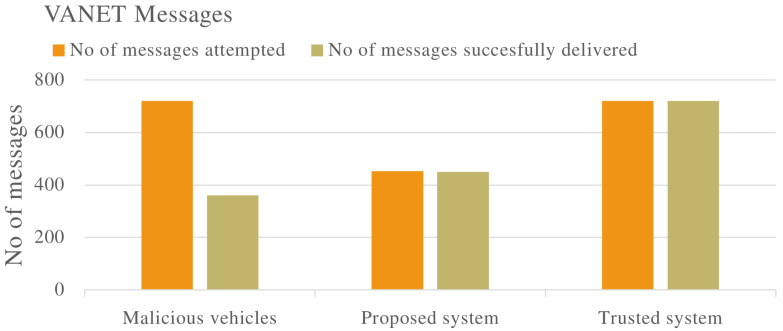
Total messages transmitted in VANET in multiple scenarios including malicious vehicles that are dropping packets in the VANET.

Figure 5 shows the number of messages attempted to be delivered, and the number of messages successfully delivered in the VANET. The VANET with non-malicious vehicles attempts and successfully delivers 720 messages. In the VANET with malicious vehicles, 720 messages are attempted while only 360 are delivered successfully. When the proposed system is applied to a VANET with malicious vehicles. Although the number of total messages attempted is less, the number of messages successfully delivered greatly improves indicating the effectiveness of the proposed system.

In the second experiment, the proposed system will be evaluated against vehicles that are exhibiting malicious behaviour of delaying messages in the VANET. A selection of vehicles will delay messages at different rates in the VANET. The results are presented.

**Figure 6:**
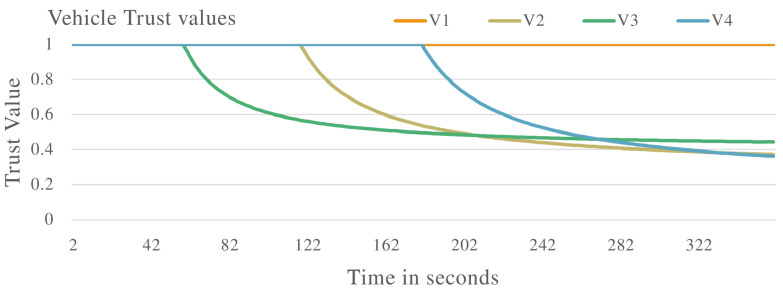
Vehicle trust values with malicious vehicles that are delaying packets in the VANET.

Figure 6 shows the vehicle trust values when the VANET is populated with vehicles exhibiting both malicious and non-malicious behaviour. V1 is identified as a non-malicious vehicle as the trust value remains constant at 1.0. V2, V3, and V4 are all identified as malicious vehicles as their trust values drop below the required threshold. These vehicles are identified to be delaying messages in the VANET.

**Figure 7:**
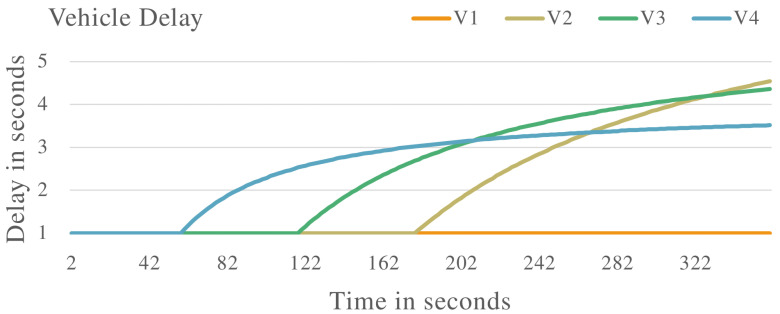
Vehicle delay with malicious vehicles that are delaying packets in the VANET.

Figure 7 shows the processing delay of the vehicles in the VANET. While V1 maintains a constant processing delay, V2, V3 and V4 start delaying packets as their processing delay increases. This shows the effectiveness of the proposed system in identifying malicious vehicles that are delaying messages in the VANET.

**Figure 8:**
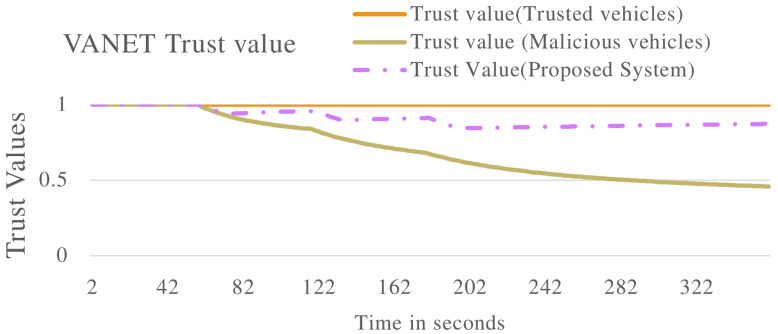
VANET trust value multiple scenarios including vehicles that are delaying packets in the VANET.

**Figure 9:**
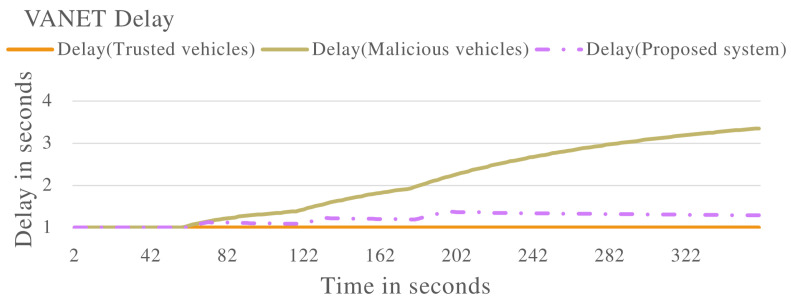
VANET delay multiple scenarios including vehicles that are delaying packets in the VANET.

Figure 8 and Figure 9 show the VANET statistics in different scenarios. The blue line, trust value (Trusted Vehicles), in Figure 8 represents the trust value of the VANET when all the vehicles are exhibiting non-malicious behaviour. The trust value remains at a constant 1.0. This is supported by the blue line in Figure 9, which represents the VANET delay when only non-malicious vehicles are present. The VANET delay is the average time taken to deliver a message in the VANET. This delay remains at a constant value of 1.0 s throughout the operation. The orange line, trust value (malicious vehicles), in Figure 8 represent the trust value of the VANET when malicious vehicles are present. The trust value drops below the threshold, showing the VANET has been taken over by malicious vehicles and can no longer perform normal operations. The orange line in Figure 9 represents the delay of the VANET with malicious vehicles, with increases consistently during VANET operation. This shows the average time to deliver messages in the VANET increases as the VANET operates. The grey line in Figure 8 and Figure 9 represents the trust value and delay, respectively, of the VANET with malicious vehicles present and the proposed system applied. It shows an improvement in the trust value of the VANET, ensuring the VANET is not taken over by malicious vehicles by isolating malicious vehicles. This in turn improves the delay of the VANET making the VANET more efficient. This proves the effectiveness of the proposed system against vehicles that are delaying messages in the VANET.

**Figure 10:**
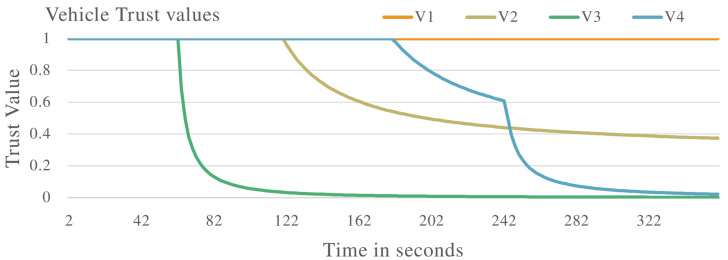
Vehicles trust values multiple malicious vehicles in the VANET that are either delaying packets, dropping packets or both delaying and dropping packets.

In the third experiment, multiple types of malicious vehicles were applied to a VANET. Malicious behaviours will include either drop packets, delay packets and or both drop and delay packets. The results are presented below. Figure 10 shows four vehicles evaluated by the proposed trust management system. V1 is identified as a vehicle exhibiting non-malicious behaviour as its trust value maintains a constant of 1.0 throughout VANET operations. V2, V3 and V4 are identified as exhibiting malicious behaviour as their trust values drop during VANET operation. These vehicles can be said to either be dropping or delaying packets in the VANET. The proposed system identifies malicious vehicles in the presence of multiple types of attacks.

**Figure 11:**
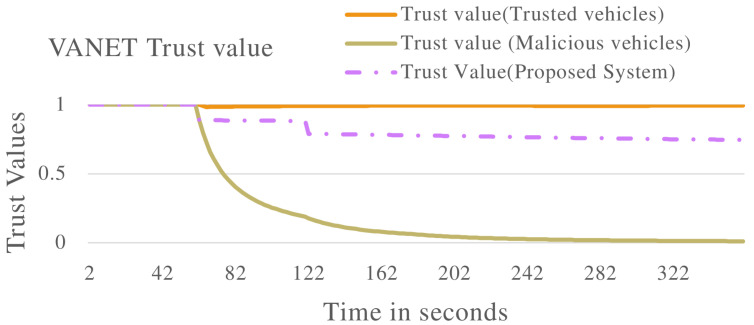
VANET trust value with multiple malicious vehicles including vehicles that are either delaying packets, dropping packets or both delaying and dropping packets.

**Figure 12:**
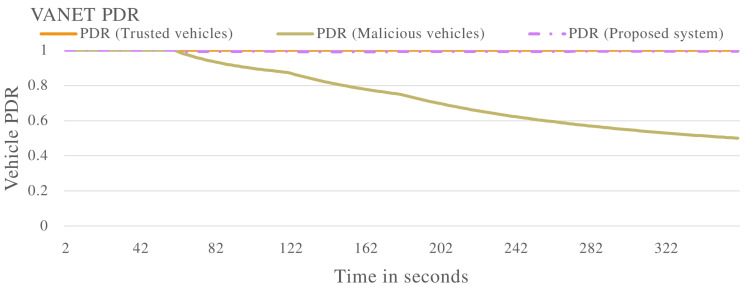
VANET packet delivery ratio with multiple malicious vehicles including vehicles that are either delaying packets, dropping packets or both delaying and dropping packets.

**Figure 13:**
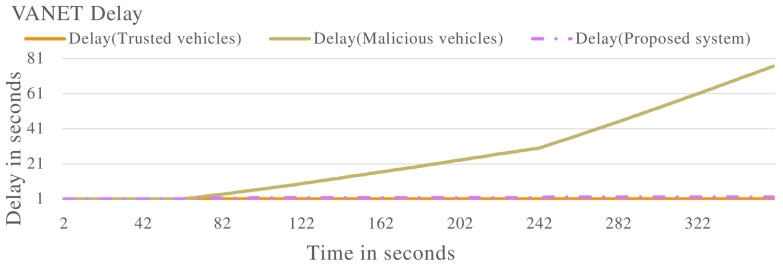
Vehicles delay multiple malicious vehicles with multiple malicious vehicles including vehicles that are either delaying packets, dropping packets or both delaying and dropping packets.

Figure 11 shows the trust value of the whole VANET with multiple types of malicious vehicles applied. The blue line in Figure 11 represents the trust value of the VANET with vehicles exhibiting non-malicious behaviour. The trust value maintains a value of 1.0 throughout operations. The orange line in Figure 11 shows the trust value of the VANET when malicious vehicles are introduced. The trust value of the VANET drops to below the trusted threshold, this indicates the VANET can no longer perform normal operations as malicious vehicles have taken over. The proposed system is introduced to a VANET with malicious vehicles, this is represented by the grey line. The proposed system is able to effectively isolate malicious vehicles, therefore the VANET remains trusted throughout the operation. This is further displayed in Figure 12 and Figure 13, where the proposed system effectively improves the packet delivery ratio and delay of the VANET.

The fourth experiment will involve testing the proposed system against network errors and false positives that may occur in the VANET. This evaluates the accuracy of the proposed system in identifying malicious and non-malicious behaviour. Network errors can cause vehicles to drop messages, or take an increased time for vehicle messages to be delivered. This can lead to false positives. False positives happen when a vehicle is identified as a malicious vehicle, yet it exhibits non-malicious behaviour. Random vehicles will be selected to simulate false positives during the operations of the proposed system. The presence of false positives should not affect the overall trust values of the vehicles. Vehicles with false positives should recover immediately if experiencing non-malicious behaviour and be identified as non-malicious.

**Figure 14:**
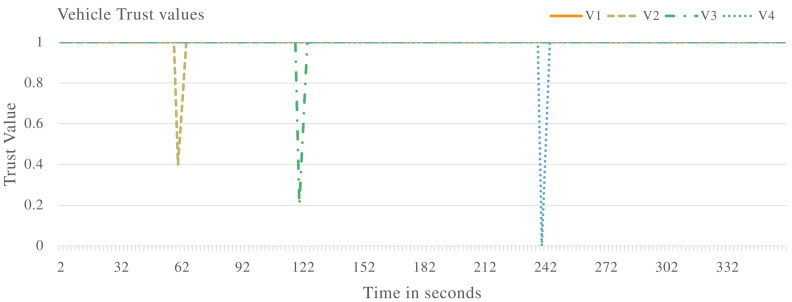
Vehicle trust value false positives.

Figure 14 displays the trust values of vehicles in the VANET. V2, V3 and V4 all experience sharp drops and rises in trust value at certain points in time. These drops can be identified as false positive reporting events. The trust value of the vehicles recovers immediately, therefore it does not affect the overall trust value of the vehicle. This improves the accuracy of the proposed system in identifying malicious and non-malicious behaviour.

## 5. Discussion

The proposed trust management system was applied to various complex scenarios and experiments. Four vehicles (V1, V2, V3 and V4) were evaluated to determine malicious or non-malicious behaviour. It was observed that the proposed system has the ability to detect malicious vehicles that are dropping and delaying messages in the VANET as shown in Figure 2, Figure 6 and Figure 10. The proposed system also improves the PDR of the VANET in the presence of malicious vehicles as shown in Figure 5 and Figure 12, although the total number of messages transmitted is reduced. The proposed system also improves the end-to-end delay of the VANET as shown in Figure 9 and Figure 13. The proposed system was also evaluated in unstable network conditions that cause false positives in the VANET, and had success in identifying false positives in the VANET as shown on Figure 14. In this paper, a multi-tier trust management system that detects malicious and non-malicious vehicles has been proposed. The RSU is responsible for calculating trust values in the VANET. A record of these trust values is kept in a ledger and used during communications in the VANET. The malicious vehicles can be isolated from important communication messages in the VANET. The proposed system also protects against watchdogs that may be colluding with malicious vehicles, e.g., in a wormhole attack. Watchdogs are selected as the most trusted vehicles in the VANET. The proposed system also protects the integrity of the calculation of the trust value. This is done by ensuring the data used to calculate trust value are legitimate data. The results show that the proposed system is successful in identifying malicious and non-malicious vehicles when applied to a VANET. The proposed system improves the VANET trust value, PDR, and delay in the presence of malicious vehicles.

The proposed system has presented some new methodologies and algorithms for determining vehicle behaviour by assigning a trust value to vehicles. The proposed system that also protects the integrity of the trust management system has been proposed. Table 3 summarises the functions of the proposed system in comparison to some trust management systems proposed for VANETs.

## 6. Conclusions and Future Work

This work has presented a research into security of VANET communications and proposed a multi-tier trust-based security system. This section will present some limitations of the study providing a direction for future research. The proposed system presented in this research was developed with a federated model, the RSUs have the responsibility of executing the algorithms presented. However, in some areas, RSUs are not densely populated. To make the proposed system more applicable and practical, it would be worthwhile to integrate the system into a cloud-based system. The algorithms and equations could be performed on a cloud system, and vehicles could query it for recommendations. The efficiency of vehicles and the VANET as a whole could benefit immensely by publishing and consuming data directly from a cloud system. A cloud-based system would also benefit the installation of the proposed system. The installation could be pushed to all vehicles and RSUs via cloud push services regardless of location. The proposed system was also applied to a VANET made up of vehicles at a stand still or moving at low speeds. Future work will involve applying the proposed to a VANET made up of vehicles moving at high speeds.

## Figures and Tables

**Table 1:** Summary of attacks.

Attack	Effect on Messages
Black Hole attack	Drop
Worm Hole attack	Drop
Message suppression/alteration	Delay
Replay attack	Both drop and delay
Timing attack	Delay
Man-in-the-middle attack	Delay
Eavesdropping attack	Delay

**Table 2:** Simulation parameters.

Parameters	Value
Area of network	200 m^2^
Number of vehicles	8
Transmission Range	20 m
Number of watchdogs	3
Initial trust value	1.0
Simulation time	360 s
Number of malicious vehicles	3
Vehicle speed	0.5 m/s
MAC protocol	IEEE802.11p

**Table 3:** Results summary.

Proposed System	Malicious Vehicle Detection	Watchdog Protection	Integrity Protection
[52]	Yes	No	No
[6]	Yes	No	No
[22]	Yes	No	No
[21]	Yes	No	No
[5]	Yes	No	No

## Data Availability

The data presented in this study are available on request from the corresponding author.

## Abbreviations

The following abbreviations are used in this manuscript:

VANET	Vehicle ad-hoc network
V2V	Vehicle-to-vehicle
V2I	Vehicle-to-Infrastructure
RSU	Road side unit
OBU	On-board unit
CCM	Central control module
TA	Trusted Authority
DSRC	Dedicated short-range communication
DOS	Denial-of-service
DDOS	Distributed denial-of-service
PDR	Packet delivery ratio
PD	Processing delay
